# Development of Water-Insoluble Vehicle Comprising Natural Cyclodextrin—Vitamin E Complex

**DOI:** 10.3390/antiox10030490

**Published:** 2021-03-20

**Authors:** Shigesaburo Ogawa, Mai Shinkawa, Ryuji Hirase, Taro Tsubomura, Katsuya Iuchi, Setsuko Hara

**Affiliations:** 1 Department of Materials and Life Science, Faculty of Science and Technology, Seikei University, Tokyo 180-8633, Japan; taiyo.mai.0823@gmail.com (M.S.); tsubomura@st.seikei.ac.jp (T.T.); sharala@h00.itscom.net (S.H.); 2 Hyogo Prefectural Institute of Technology, 3-1-12 Yukihira-cho, Suma, Kobe 654-0037, Japan; hirase@hyogo-kg.jp

**Keywords:** vitamin E, *γ*-cyclodextrin, vehicle, radical scavenging, oxidative stress, cell viability

## Abstract

Development of a novel antioxidant-delivery vehicle exerting biosafety has been attracting a great deal of interest. In this study, a vehicle comprising a natural composite consisting of vitamin E (α-tocopherol; Toc) and cyclodextrin (CD) additives was developed, directed toward aqua-related biological applications. Not only *β*-CD, but also *γ*-CD, tended to form a water-insoluble aggregate with Toc in aqueous media. The aggregated vehicle, in particular the *γ*-CD-added system, showed a remarkable sustained effect because of slow dynamics. Furthermore, a prominent cytoprotective effect by the *γ*-CD–Toc vehicle under the oxidative stress condition was confirmed. Thus, the novel vitamin E vehicle motif using *γ*-CD as a stabilizer was proposed, widening the usability of Toc for biological applications.

## 1. Introduction

Vitamin Es are representative lipophilic antioxidants, consisting of a chromanol head and a phytyl tail [1,2,3,4,5,6,7,8,9]. It is well-known that the molecules play a crucial role in the treatment of free radicals-induced diseases, so-called oxidation-induced diseases, including cardiovascular diseases [2], neurodegenerative diseases [3,4], as well as inflammatory diseases [5,6]. In the treatment, vitamin E can interfere with one or more propagation steps of the lipid peroxidation process and thus minimize oxidative stress.

The low solubility and dispersibility of vitamin E in water media have been typically shown [1,7,8,9]. To address the issue, the exploration of innovative formulation technology has been eagerly investigated. An attractive techniques is the use of dispersible substrates in water media, which increase bioavailability, and enable targeted delivery of antioxidants, as well as temporally controlled release at the site of action [7]; prolonged and sustained drug release were desired to release the targeted delivery of the antioxidants. Biocompatible polymers, such as chitosan and casein, have been reported as substrates which encapsulate vitamin E and enhance its bioavailability [7,10]. On the other hand, in this study, natural cyclodextrins (CDs), which are water-soluble, were used as a substrate to develop a novel “water-insoluble” vitamin E vehicle in water media.

**Figure 1:**
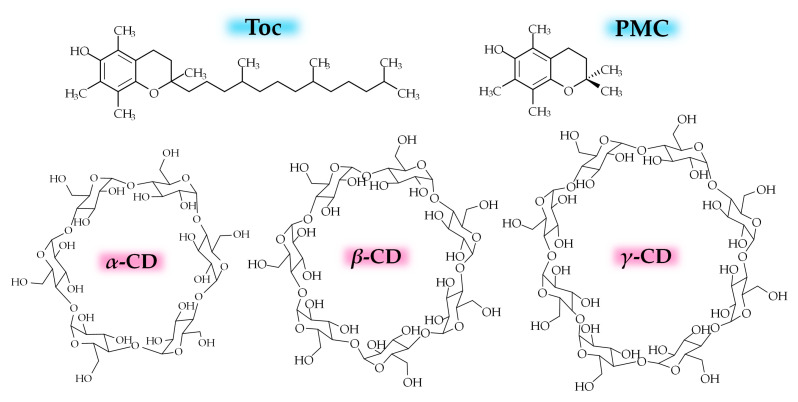
Molecular structures of (blue) a vitamin E (*α*-Tocopherol; Toc), a water-soluble artificial Toc analogue, 2,2,5,7,8-pentamethyl-6-chromanol (PMC), and (pink) natural cyclodextrin (CD) compounds used in this study.

CD is a cyclic oligosaccharide that consists of glucose residues linked by an α-1,4 glycosidic bond [11,12,13,14]. *α*-CD, *β*-CD, and *γ*-CD are the representative natural CDs that consist of six, seven, or eight glucose residues, respectively, and are used in various medical applications (Figure 1). CDs can form inclusion complexes with various hydrophobic guest molecules using a hydrophobic internal cavity, thereby typically affording additional properties, such as solubility, stability, and availability [11,12,13,14]. Regarding CD–vitamin E systems, encapsulation has been successfully applied in the development of assay techniques to measure the antioxidant activity of different compounds in the presence of water [15,16,17]. In addition, several fascinating antioxidant activities of vitamin E (*α*-Tocopherol; Toc; Figure 1) complexed with CDs in water/organic solvent mixed systems, including cream preparations (oil-in-water emulsions) [18,19], propylene glycol/water mixtures [18], low-density polyethylene films [20], chloroform/methanol mixtures [21], methanol/water (1:1) mixtures [22] and methanol [23,24] have been reported. In some of these cases, the controlled release of Toc to the lipid phase and an organic solvent or from a polymer film has been described [20,21,22,23,24]. However, these studies used an organic solvent, and, to the best of our knowledge, no sustained release of the radical scavenging function of Toc has been reported for CD–Toc complex systems in water media in the absence of an organic solvent, which is needed for biological applications.

Recently, we have studied the solubility enhancement of Toc by CDs [25]. As a result, it was found that 2,6-di-O-methylated *β*-CD (2,6-DMCD) greatly enhanced the solubility of Toc, whereas the natural CDs, such as *β*-CD and *γ*-CD, which are also water-soluble CDs, formed opaque systems by mixing with Toc. From the preliminary study, we deemed that the dispersion systems can be used as antioxidant-delivery vehicles in biological applications. Both components, Toc and CD, have been used as food ingredients and pharmaceutical excipients [12], and therefore, the exertion of biosafety could be expected.

Herein, we describe the effects of natural CDs on dispersibility with Toc in water media. The unique preparation conditions, in particular for *γ*-CD–Toc systems, were investigated in detail. Furthermore, the radical scavenging ability in water systems was evaluated, together with an assessment of the cytoprotective effect on cells under oxidative stress using a lipid oxidation inducer. These studies were performed in the absence of an organic solvent. Throughout the study, 2,2,5,7,8-pentamethyl-6-chromanol (PMC, Figure 1) was used for investigating the interaction of the chromanol head with CD and as a reference for the radical scavenging test. As a result, a novel vehicle consisting of *γ*-CD and Toc with potential for biological applications is proposed.

## 2. Materials and Methods

### 2.1. Materials

Toc (MW: 430.71), and PMC (MW: 220.31) were purchased from Fujifilm Wako Pure Chemical Industries, Ltd. (Osaka, Japan). *α*-CD, *β*-CD, and *γ*-CD were obtained from TCI Co. Ltd. (Tokyo, Japan). 2,2′-Azinobis (3-ethylbenzothiazoline-6-sulfonic acid ammonium salt) (ABTS) (>98%, MW: 548.67) was purchased from TCI Co. Ltd. (Tokyo, Japan).

### 2.2. Cell Culture and Treatment of Cell with Chemicals

The African green monkey kidney cell line COS-7 (JCRB9127) was cultured in Dulbecco’s Modified Eagle’s Medium supplemented with 10% fetal bovine serum (GE Healthcare Life Sciences, Tokyo, Japan), penicillin (Nacalai Tesque, Kyoto, Japan), streptomycin (Nacalai Tesque), and amphotericin B (GE Healthcare Life Sciences, Tokyo, Japan). Cells were seeded into wells of 96-well or 24-well plates at a concentration of approximately 0.3 cells/mL, in 100 or 500 µL per well, respectively, and plates were incubated for 24 h at 37 °C in an atmosphere containing 5% CO_2_. Cells were then cultured in the presence or absence of *γ*-CD (60 µM) or *γ*-CD (60 µM)-Toc (10 µM) for 30 min under identical incubation conditions, before exposure to 0, 50, 100, or 200 µM *tert*-butyl hydroperoxide (TBHP) (an inducer of lipid peroxidation; B2633, Sigma-Aldrich, Tokyo, Japan) [26]. Cells were cultured under identical incubation conditions for a further 18 h.

### 2.3. Preparation and Characterization of CD and Toc Mixtures

#### 2.3.1. Preparation of Sample I

To prepare the CD-Toc mixture system, an excess amount of Toc or the Toc analogue PMC was added to 4 mL of an aqueous CD solution at different concentrations. Stirring at ambient atmosphere was conducted for 16 h. It was found that some amount of added Toc adhered to the Teflon magnetic bar or the glass wall during stirring (Figure S1, Supplementary Materials), and thus not all the Toc could be successfully dispersed in water. Since the nonstabilized Toc by CD tended to contact with solid surfaces, the homogeneous dispersion was obtained by several moving operations to other glass containers containing magnetic bar to afford dispersion I.

#### 2.3.2. Preparation of Sample II

II-1:4: CD-Toc dispersion systems were prepared using the CD-Toc mixing solids [25,27]. Briefly, Toc (0.23 mmol) in 5 mL EtOH and CDs (0.93 mmol; 4 times of Toc) in 10 mL pure water were mixed, and solid inclusion complexes were obtained by co-precipitation under stirring at 8000 rpm for 3 min using an ultra-dispenser (LK-22, Yamato Scientific Co. Ltd., Tokyo, Japan). Then, the solvent was evaporated under reduced pressure at high temperature. Here, the highest temperature of the water bath was 45 °C and the sample was always protected from light. Each solid sample was stored under an N_2_ gas atmosphere in the dark. The dispersion was prepared by the addition of water and subsequent mixing with vortex and ultrasonication within 1 min, respectively.

II-1:2: Toc (0.46 mmol) in 5 mL EtOH and CDs (0.93 mmol; 2 times of Toc) in 10 mL pure water were also used for the preparation of dispersion II-1:2 to demonstrate the effectiveness of the *γ*-CD-added system.

#### 2.3.3. Determination of Toc Concentration

The Toc concentration of each sample was determined spectrophotometrically at 291 nm using a V-650 spectrophotometer, JASCO Corp. (Tokyo, Japan) [22,23,24,25]. To prepare the dissolved samples for spectrophotometric analysis, DMSO was used for dissolution and dilution.

#### 2.3.4. Characterization of Solid Used for the Preparation of II

The analysis of the compositions in the solid samples, used for the preparation of II, was performed by NMR spectroscopy using a JEOL ECA 500 instrument (Tokyo, Japan). The physicochemical properties of the solids were analyzed by Fourier transform infrared (FTIR) using a Nicolet iS50+iN10 (Thermo Fisher Scientific Inc., WI, USA). To obtain novel insight into the physical properties, emission quantum yields in solid states were evaluated using a combination of an integrating sphere (Labsphere, Model 4P-GPS-030-SF, NH, USA), a monochromated xenon light source, and a cooled CCD spectrometer.

### 2.4. Radical Scavenging Ability Test in Water

For the radical scavenging test, an ABTS biradical was used similar to previous works [25,28]. Namely, ABTS (117.8 mg) was dissolved in water (30.6 mL), and potassium persulfate (28.2 mg) was then added to activate the ABTS radical. The solution was stored overnight in the dark. The ABTS radical solution was diluted to the appropriate concentration for spectroscopic measurement. An ABTS radical scavenging test was performed by adding each CD dispersion to the aqueous ABTS solution. The absorption at 742 nm was measured. As a reference, an aqueous PMC solution in the absence of CD was used. For the investigation on the dispersion systems, the baseline of the sample system without ABTS was subtracted from the result for the tested sample with ABTS.

### 2.5. Assessment of Cell Viability and Morphology

Cell viability was assessed using a Cell Counting Kit-8 (CCK-8; Dojindo Laboratories, Kumamoto, Japan), according to the manufacturer’s protocol. Briefly, CCK-8 reagent was added to cells, which were then incubated for 2 h at 37 °C in an atmosphere containing 5% CO_2_. Optical density at 450 nm was measured using a Food Mark microplate absorbance reader (Bio-Lad, CA, USA). Cell morphology was assessed using a DM IL LED microscope (Leica Microsystems, Wetzlar, Germany).

### 2.6. Statistical Analysis

All statistical analyses were conducted using Excel 2016. All data are presented as the mean ± standard deviation (SD) of four independent experiments. Between-group differences were compared using Student’s *t*-test. Differences were considered statistically significant at * *p* < 0.05. Levels of statistical significance for each comparison are indicated in each figure.

## 3. Results

### 3.1. Investigations on Dispersion Behaviours of CD and Toc Mixtures

**Figure 2:**
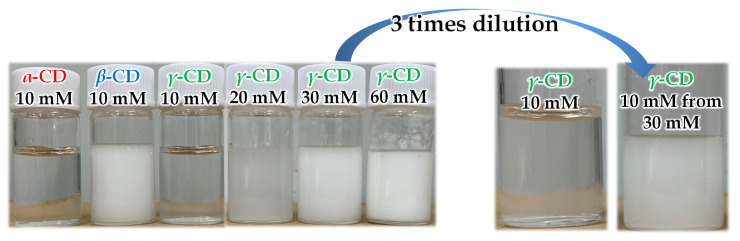
Photographs for I and for a sample diluted from 30 mM to 10 mM of *γ*-CD, respectively.

When stirring a water-soluble CD solution at 10 mM concentration with a water-insoluble vitamin E (Toc), an increase in turbidity was observed for each CD-added system, especially for the *β*-CD system (Figure 2). The dispersion for the *β*-CD system contained a large amount of Toc of about 2.6 mM, whereas the amounts of Toc stabilized by *α*-CD and *γ*-CD were negligible under the condition. However, in the case of *γ*-CD, the turbidity drastically increased with the initial concentration of *γ*-CD above 20 mM. The dispersions prepared with *γ*-CD concentrations above 20 mM were stable even after the dilution to 10 mM (Figure 2, right), whereas the dilution of the dispersion of *β*-CD system destabilized rapidly (i.e., the five times dilution of the *β*-CD system (10 mM) with a vortex mixing afforded a distinct agglomeration within 10 min). The results indicated the high stability of the dispersion formed in *γ*-CD-added systems.

Noteworthy, when transparent solutions were obtained by the filtration of these dispersions through a 0.2 μm nanopore membrane filter, the Toc contents in the *β*-CD-, and *γ*-CD-added systems became trace. This indicates the formation of large aggregates containing Toc for the *β*-CD- and *γ*-CD-added systems, which were insoluble in water.

**Figure 3:**
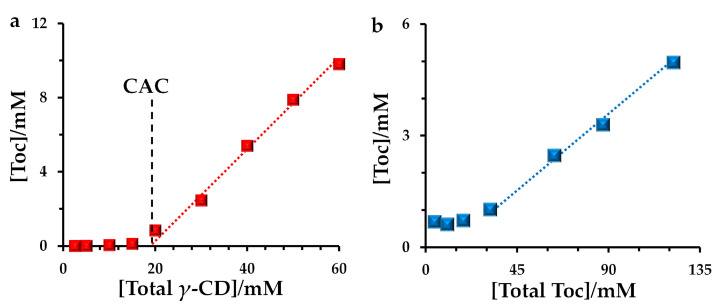
Contained vitamin E (Toc) concentration in I as the function of (**a**) initial *γ*-cyclodextrin (CD) concentrations and (**b**) added total amount of Toc. For (**a**), the added amount of Toc was 1.2 times mole of *γ*-CD. For (**b**), the *γ*-CD concentration was constant at 30 mM.

Figure 3a shows the unique concentration dependence of *γ*-CD. The amount of Toc suddenly increased above 20 mM *γ*-CD concentrations for I, suggesting that the concentration can be regarded as the critical aggregation concentration (CAC) to afford *γ*-CD/Toc-dispersions. As far as we know, the observation of CAC for CD/Toc systems was unprecedented. Furthermore, as shown in Figure 3b, the amount of contained Toc increased as the increase of the amount of total Toc was added for the preparation. However, most of the Toc remained in a phase-separated state for the preparation (Figure S1, Supplementary Materials), and only one-thirtieth of the Toc could be used. A nonlinear increase was observed in Figure 3b at the low Toc amount conditions, possibly owing to the adhesion problem of Toc on the surfaces of magnet and/or glass, respectively.

The dispersion states could be prepared using co-precipitated samples. A stable dispersion was obtained for II-1:4 of the *γ*-CD-added system, whereas those of the *α*-CD-added and *β*-CD-added systems were unstable (Figure S2). After stirring for 16 h, only trace amounts of Toc were dispersed for *α*-CD-added and *β*-CD-added systems, but the ideal amount of Toc still remained in the homogeneous dispersion for the *γ*-CD-added system (Figure S2b). Noteworthy, the *γ*-CD-added system afforded high stability even for its II-1:2 sample with the high Toc ratio. The lower the concentration became, the aggregate with smaller size tended to form (Figure S3). These results showed the concentration-dependent aggregation behavior of *γ*-CD/Toc dispersion.

### 3.2. Discussion on Correlations of Dispersibility and Inclusion Complex Formation of CD/Toc Mixtures

With regard to the CD-Toc systems, previous investigations have shown the formation of inclusion complexes for *β*-CD-Toc systems in the solid state [27]. On the other hand, the poor complexation capacity was expected for *α*-CD because it does not complex with the phytyl chain of Toc owing to its small pore size [29]. The excellent complexation ability of 2,6-DMCD and the phytyl chain and chromanol ring was confirmed in the solution by 2D ROESY NMR [25]. Since the hole size order of CD was *γ*-CD > *β*-CD ≈ 2,6-DMCD > *α*-CD [13], the hole size of *γ*-CD should be sufficient to complex with the phytyl chain and chromanol ring. In addition, in terms of the possibility of the complexation between CD and chromanol head, we tried to obtain the information using PMC as a substituted substrate without a phytyl chain. Hence, the solubility enhancement of PMC was studied in the presence of CDs. The enhancement of the solubility was considered as a result of the formation of an inclusion complex.

As shown in Figure S4 in the Supplementary Materials, both *β*-CD and *γ*-CD showed the apparent solubility enhancement of PMC, whereas slight increases were observed for *α*-CD-added systems in the concentration range from 0 to 60 mM of *α*-CD; Because of the low solubility of *β*-CD in water [13], a narrow concentration range was investigated for the *β*-CD-added system. It indicated that both *β*-CD and *γ*-CD can form the complex via the interaction with a chromanol ring, whereas *α*-CD cannot form the complex. This fact supported that both *β*-CD and *γ*-CD can form a complex with Toc via the interaction with chromanol ring, whereas *α*-CD was assumed not to form a stable inclusion complex because it cannot strongly interact with both the phytyl chain and the chromanol ring, respectively. Considering it, a clear correlation was found. Namely, *β*-CD and *γ*-CD can form an inclusion complex with Toc and stabilize the insoluble dispersion, whereas no distinct dispersion was obtained for the *α*-CD-added system because of the low complexation ability with Toc.

Noteworthy, as observed in some organic solutions [30], the high quantum yield of fluorescent by Toc was observed for the solid mixture with *γ*-CD, whereas much lower values were obtained for *α*-CD and *β*-CD-added mixtures (Figure S5, Supplementary Materials). Because no fluorescence was observed in pure Toc matrix in bulk, we regarded that *γ*-CD and Toc mixture showed strong affinity between each other and most of each component must contribute to the formation of stable inclusion complex in the solid, thus affording the stable quantitative mixture when dispersed. In the FTIR spectrum for the *γ*-CD-added solid prior to dispersing, no apparent intense bands were confirmed at 2924 and 2867 cm^−1^ for asymmetrical methylene and symmetrical methyl stretching vibration in the Toc molecules (Figure S6, Supplementary Materials). It has been proposed as one of the evidence of the formation of inclusion complex for CD–Toc mixture [27].

From these results, it was considered that the characteristic dispersing behavior of the CD–Toc systems resulted from the self-assembly of the inclusion complex formed between CD and Toc.

### 3.3. ABTS Radical Scavenging Ability of the Toc and CD Mixtures

**Figure 4:**
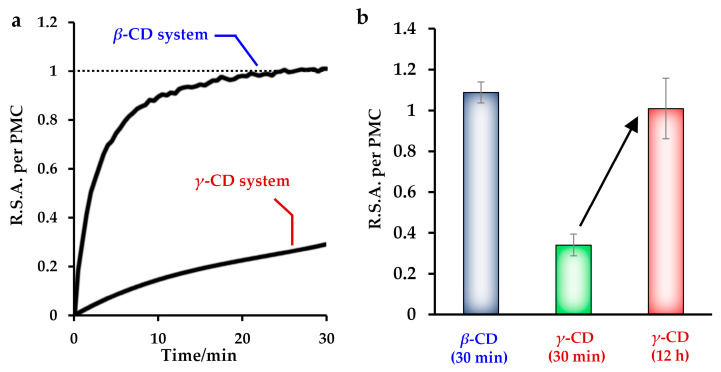
Results of the ABTS radical scavenging test for sample I. (**a**) Time dependence measurement and (**b**) radical scavenging ability (RSA) after 12 h and 30 min for the *γ*-cyclodextrin (CD)-added systems. The *β*-CD and *γ*-CD concentrations were diluted from 10 mM and 30 mM for the test, respectively, and the measurement was performed for the CD concentrations below 0.8 mM.

Next, a radical scavenging test was conducted in water. Each radical scavenging ability (RSA) of Toc in mol was expressed as the scavenging ability of PMC in mol, which was evaluated in the water system in the absence of both organic solvent and CD and used as a reference (unity). It should be noted that the addition of CDs does not affect the RSA of PMC at the concentrations used for the study. The representative results of the RSA test are shown in Figure 4.

RSA was observed when Toc was contained in the sample. As shown in Figure 4a, CD–Toc dispersions prepared from 10 mM *β*-CD and 30 mM *γ*-CD aq showed effective RSA under diluted conditions. Noteworthy, while the 2,6-DMCD-added system showed no sustained effect in the previous study [25], slight and large sustained effects were observed for the *β*-CD-added and *γ*-CD-added systems, respectively. This means that the dispersed Toc stabilized by *β*-CD and *γ*-CD, especially in the latter case, showed limited access owing to the hindered molecular diffusion of the self-assembled aggregates. Similar behavior was observed for sample II prepared using an inclusion solid consisting of *γ*-CD and Toc (Figure S7, Supplementary Materials).

In addition, when the reaction was complete, the RSA reached about unity (Figure 4b), meaning that the Toc stabilized by CD showed a comparable radical scavenging effect to that of the corresponding PMC system. This sustained and effective radical scavenging are unique features for vehicles for biological applications, in which water-soluble PMC and Trolox cannot be used.

### 3.4. Cytoprotective Activity of the γ-CD–Toc Complex in Cultured Cells

As described, unique properties of the *γ*-CD–Toc complex include its ability to self-assemble, and a high and very slow RSA. Then, the cytoprotective impact of the *γ*-CD–Toc complex on TBHP-induced oxidative stress-mediated cell damage [26] was investigated in vitro.

**Figure 5:**
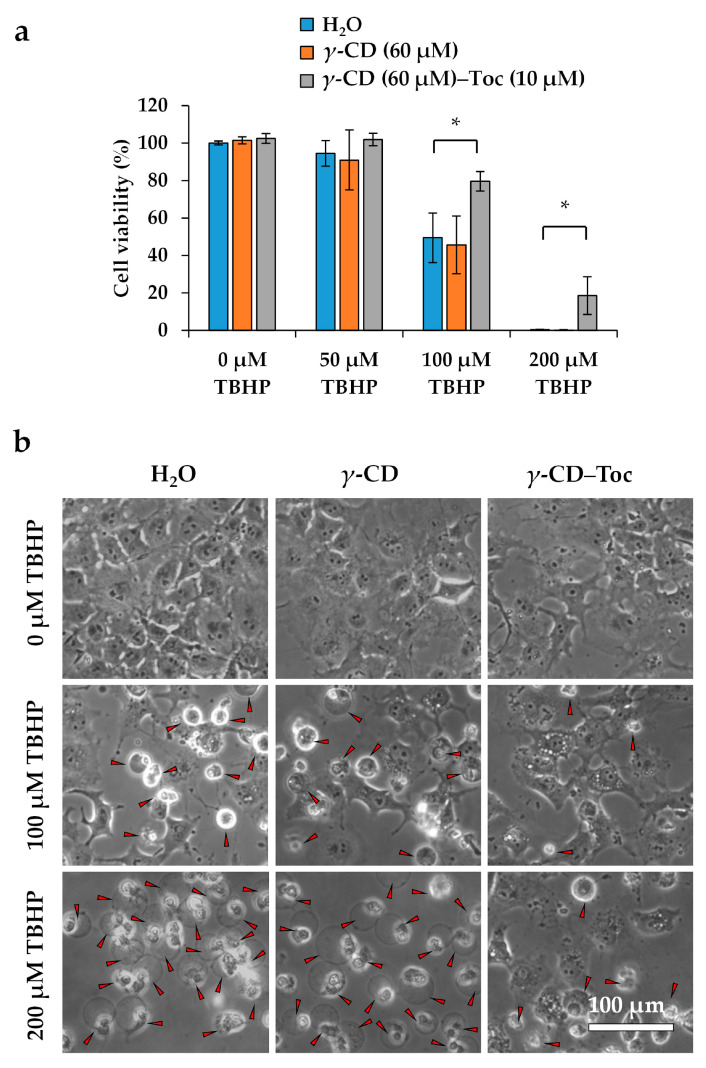
Cytoprotective impact of the *γ*-cyclodextrin (CD)-vitamin E (Toc) complex in the presence of a cytotoxic oxidative stressor. (**a**) Viability of COS-7 cells cultured for 30 min in the presence or absence of *γ*-CD or *γ*-CD-Toc before exposure to 0, 50, 100, or 200 µM *tert*-butyl hydroperoxide (TBHP) for 18 h. Cell viability was measured using a Cell Counting Kit-8 (Dojindo Laboratories) (* *p* < 0.05). (**b**) Phase-contrast photomicrographs of COS-7 cells cultured for 30 min in the presence or absence of *γ*-CD or *γ*-CD-Toc before exposure to 0, 100, or 200 µM TBHP for 18 h. The red arrowhead indicates a dead cell.

Exposure of COS-7 cells to TBHP induced loss of viability in a dose-dependent manner (Figure 5a) and resulted in altered cellular morphology (Figure 5b). Pretreatment with *γ*-CD–Toc significantly ameliorated the cytotoxic effect of higher TBHP doses (100 and 200 µM), as demonstrated by improved cell viability and preserved cell morphology (Figure 5). The *γ*-CD–Toc complex exhibited more potent cytoprotective activity than *γ*-CD alone. These data suggest that the *γ*-CD–Toc complex enters COS-7 cells and scavenges intracellular lipid radicals in a cytoprotective manner. Thus, this complex may provide utility not only for molecular biological research, but also perhaps as a novel therapeutic agent for clinical use in the context of oxidative stress-associated diseases.

## 4. Conclusions

The formation of CD–Toc inclusion dispersion and the effects on the radical scavenging and the cytoprotection were demonstrated. The mixture of *γ*-CD and Toc formed a highly stable dispersion when the initial CD concentration was above 20 mM or when it was prepared using inclusion complex solids obtained via the co-precipitation method. On the other hand, the mixture of *β*-CD and Toc also formed a stable dispersion when the initial CD concentration was 10 mM, but the system lost the dispersing state under dilution unlike the dispersion formed in the *γ*-CD-added system, meaning that the dispersion formed in the *γ*-CD-added system must be more stable and useful.

Several studies including the phase solubility profile of CD–PMC systems and the spectroscopic analysis on the solid consisting of CD and Toc indicated the correlations between the characteristic dispersing behavior of the CD–Toc systems and the self-assembly of inclusion complex formed between CD and Toc. Detail investigations on the formation of mechanisms at the molecular level were required as future researches.

Noteworthy, most of the inclusion complexes were not solubilized, but the CD–Toc self-assembly dispersion systems showed a radical scavenging effect comparable to that of PMC. In particular, the *γ*-CD–Toc system exhibited a distinct packaging effect, and a sustained effect was obtained. Moreover, the effectiveness of *γ*-CD–Toc inclusion assembly was confirmed for the cytoprotective activity in vitro. These results showed unique fascinating features of *γ*-CD–Toc inclusion assembly for novel antioxidant-delivery-vehicles for biological applications, in which water-soluble analogues, PMC and Trolox, cannot be used. The use as a therapeutic agent for clinical use in the context of oxidative stress-associated diseases was expected.

## Data Availability

Data is contained within the article or supplementary material.

## Supplementary Materials

The following are available online at https://www.mdpi.com/2076-3921/10/3/490/s1, Figure S1: A photograph showing adhered Toc on Teflon magnet bar, Figure S2: Photographs and contained Toc concentrations of samples of II-1:4, Figure S3: Photographs and schematic illustration of the concentration-dependent behavior of Sample II-1:2 of *γ*-CD/Toc mixture, Figure S4: Phase solubility profile of CD/PMC mixtures, Figure S5: Fluorescent quantum yield of Toc in the co-precipitated solids, Figure S6: FTIR spectrum of the co-precipitated *γ*-CD/Toc solids, Figure S7: Time dependence measurement of ABTS radical scavenging test for II-1:4 of *γ*-CD/Toc mixture.
