# Supplementary material for: Development of Water-Insoluble Vehicle Comprising Natural Cyclodextrin—Vitamin E Complex
Source: Antioxidants (Basel). 2021 Mar 20;10(3):490. doi: 10.3390/antiox10030490 (PMC8003986; doi:10.3390/antiox10030490)
Supplement: Supplementary file 1 [file antioxidants-10-00490-s001.pdf]

Supplementary Materials

Development of water insoluble vehicle comprising natural cyclodextrin–vitamin E complex

Shigesaburo Ogawa [1,*], Mai Shinkawa [1], Ryuji Hirase [2], Taro Tsubomura [1], Katsuya Iuchi [1,*], and Setsuko Hara [1]

[1] Department of Materials and Life Science, Faculty of Science and Technology, Seikei University, Tokyo 180-8633, Japan

[2] Hyogo Prefectural Institute of Technology, 3-1-12 Yukihira-cho, Suma, Kobe, 654-0037, Japan

* Corresponding author: s.ogawa.chem@gmail.com; iuchi@st.seikei.ac.jp

Table of Contents

1. Supplementary information relating to sample preparation..S2

2. Discussion on inclusion complex formation.. S4

3. Radical scavenging test for co-precipitation solid..S5

1. Supplementary information relating to sample preparation

1.1. Problematic issue of sample preparation

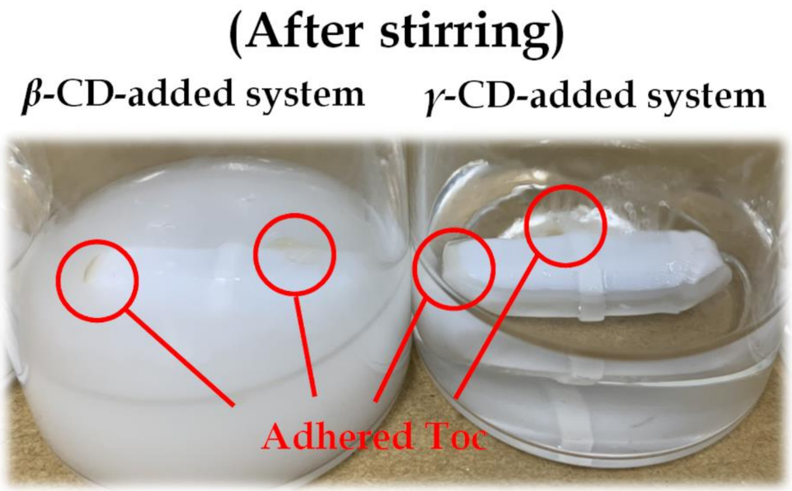

Figure S1. A photograph showing adhered Toc onto the Teflon magnetic bar in the sample mixture of 15 mM Toc and 10 mM aqueous CDs solution (Left: ***β*-CD-added system**, Right; ***γ*-CD-added system**).

1.2. Sample II-1:4

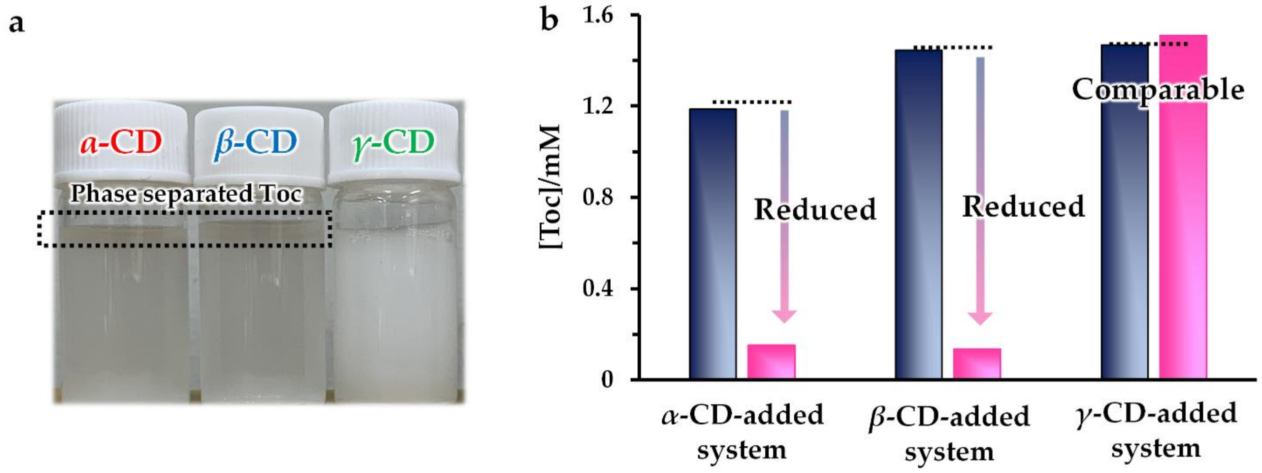

Figure S2. (a) Photographs and (b) contained vitamin E (Toc) conc. [mM] for the sample II-1:4, in 10 wt% of cyclodextrins (CDs)/Toc mixtures. For (b), the left bars showed the ideal Toc concentrations under the estimation that all Toc could be dispersed and the right bars showed the practical values in the homogeneously dispersed portion. The phase separation as observed in (a) reduced the Toc content in the dispersion as shown in (b).

1.3. Sample II-1:2

The dispersion of II-1:2 from the solids of γ-CD/Toc mixture showed apparent concentration dependent aggregation behavior (Fig. S3). For instance, when the 1.0 wt% and 0.5 wt% dispersing systems, the precipitation was apparently observed in each system, whereas no such precipitation was confirmed for 0.15 wt% dispersion system. Moreover, when the dispersing particles were filtrated through a 1.0 μm nanopore membrane filter, the persistence of nano-dispersion states with semitransparency was confirmed for 0.15 wt% and 0.5 wt% samples, respectively. It means that the small sized aggregate formed in the low concentration system, for the degree of semitransparency have increased with the decrease of the concentration of γ-CD/Toc mixture. Thus, the schematic image as shown in right of Fig. S3 was described.

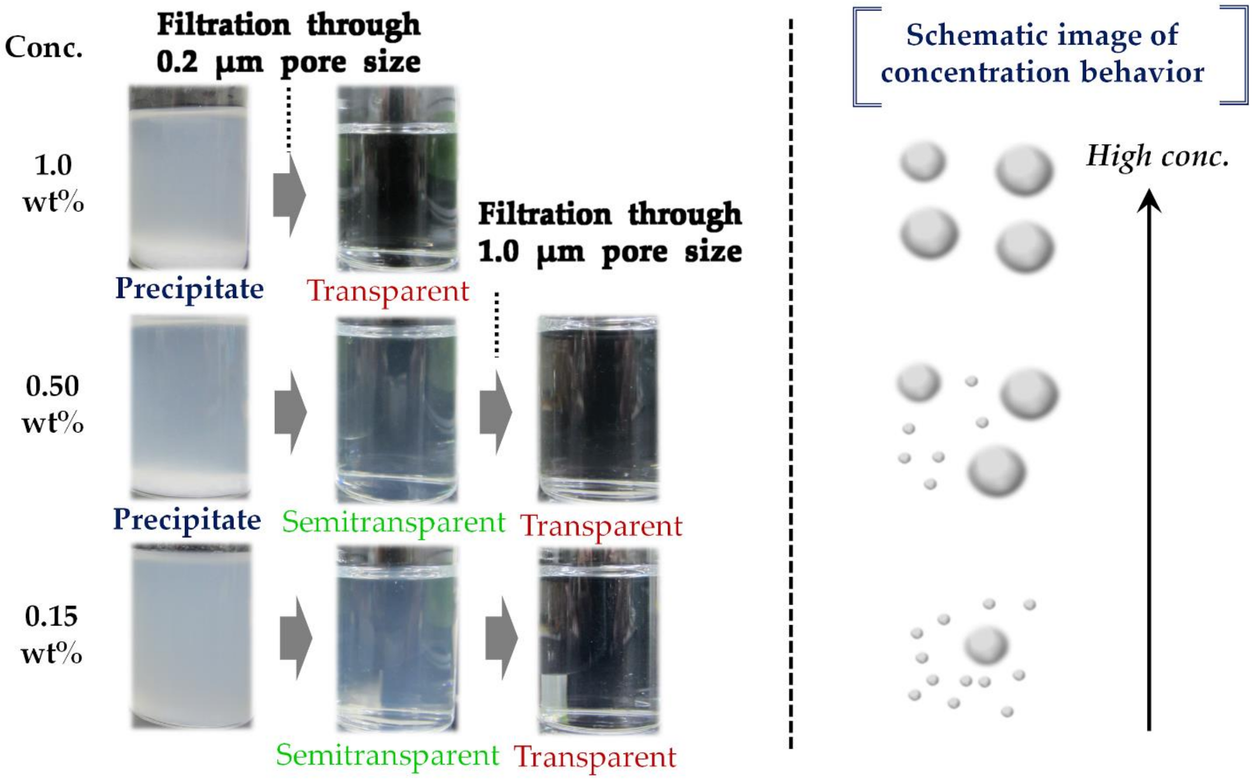

Figure S3. Photographs and schematic image of concentration–dependent aggregation behaviour for γ-cyclodextrin (CD)–vitamin E (Toc) mixture.

2. Discussion on inclusion complex formation

2.1. Phase solubility test of CD–PMC systems

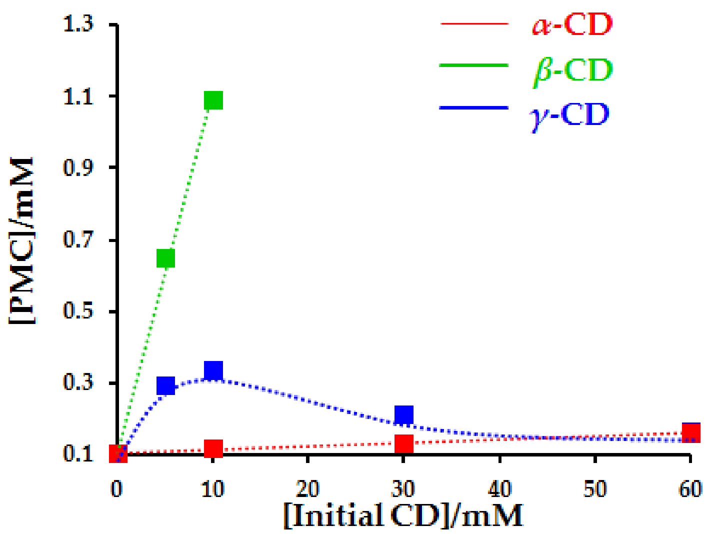

Figure S4. Phase solubility diagram of the cyclodextrin (CD)–2,2,5,7,8-pentamethyl-6-chromanol (PMC) system. The increase of PMC concentration indicated the formation of inclusion complex between the two components.

2.2. Emission property of co-precipitated solids consisting Toc and CD

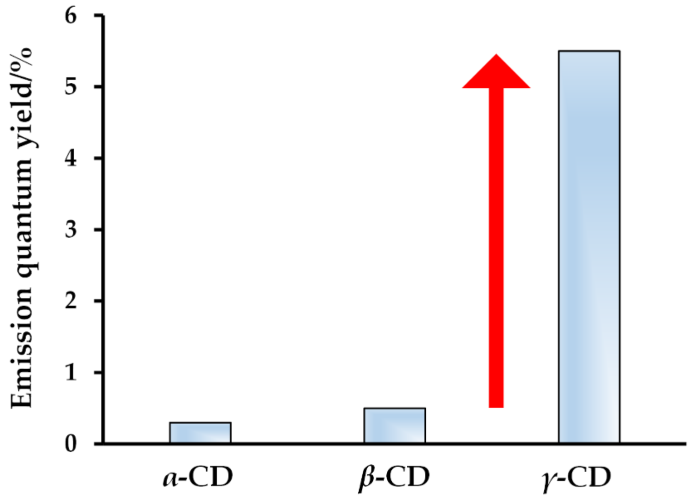

Figure S5. Fluorescent quantum yield of Toc in the co-precipitated solids (Charged vitamin E (Toc):cyclodextrin (CD) ratio = 1:4). Only the γ-CD/Toc solid showed a high quantum yield owing to the formation of stable inclusion complex.

2.3. FT-IR spectrum of the co-precipitated solid

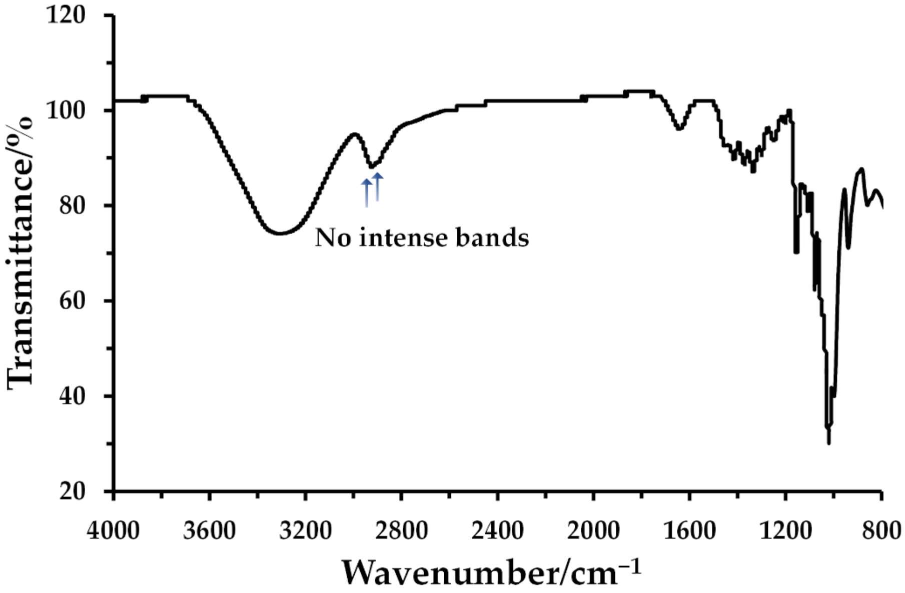

Figure S6. FT-IR spectrum of the co-precipitated γ-cyclodextrin(CD)/vitamin E(Toc) solid (Charged Toc:CD ratio = 1:2).

3. Radical scavenging test for co-precipitation solids

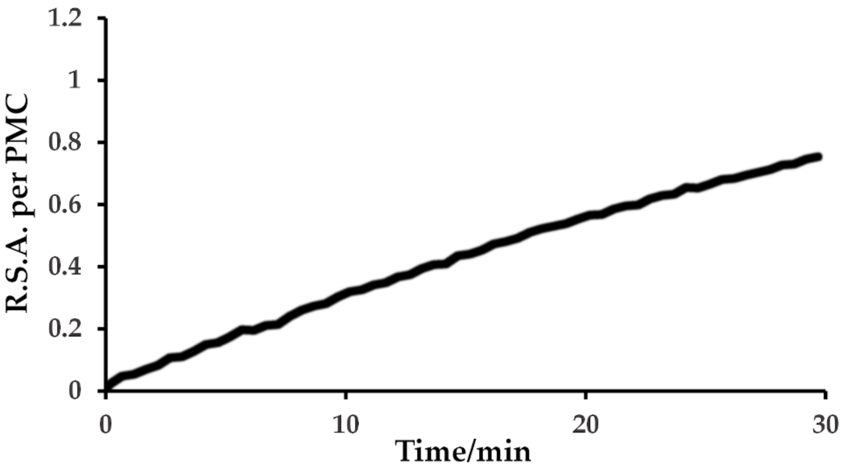

Figure S7. Time dependence measurement of ABTS radical scavenging test for II-1:4 of γ-CD–Toc mixture.
